# Reducing mortality in hip fracture patients using a perioperative approach and “Patient- Centered Medical Home” model: a prospective cohort study

**DOI:** 10.1186/1754-9493-8-7

**Published:** 2014-02-03

**Authors:** Jove Graham, Thomas R Bowen, Kent A Strohecker, Kaan Irgit, Wade R Smith

**Affiliations:** 1 Geisinger Center for Health Research, 100 N. Academy Ave., Danville PA 17822, USA; 2 Department of Orthopedics, Geisinger Health System, Danville, PA, USA; 3 Mountain Orthopaedic Trauma Surgeons at Swedish, Englewood, CO, USA

**Keywords:** Hip fracture, Mortality, Care coordination, Primary care

## Abstract

**Background:**

Hip fracture patients experience high morbidity and mortality rates in the first post-operative year after discharge. We compared mortality, utilization, costs, pain and function between two prospective cohorts of hip fracture patients, both managed with identical perioperative protocols and one group subsequently managed via a “Patient-Centered Medical Home” (PCMH) primary care management model.

**Methods:**

We analyzed 6 and 12-month outcomes from two matched cohorts of patients who were surgically treated for hip fracture from January 1, 2010 to June 30, 2011 at two hospitals (n = 194). Controls did not receive PCMH and were matched to cases on surgery date, sex, age, and comorbidities. Mortality and healthcare utilization were the primary outcomes studied, with medical costs, quality of life, pain and function at 12 months assessed as secondary outcomes in a subgroup. Survival analysis, regression and Student-t testing were used with p < 0.05 considered significant.

**Results:**

At 6 months, PCMH patients had significantly lower mortality than patients receiving standard care (11% vs. 26%, p < 0.01). At 12 months, a difference persisted (23% vs. 30%, p = 0.12) but was no longer statistically significant. Mean quality of life scores were similar (0.73 vs. 0.76, p = 0.49) and Harris Hip score was slightly improved for PCMH (73 vs. 64, p = 0.04). Mean costs per patient per month were lower for PCMH but not significantly different ($69 vs. $141, p = 0.20 for pharmacy costs; $1212 vs. $1452, p = 0.45 for non-pharmacy costs).

**Conclusions:**

Patients receiving aggressive post-discharge care from a PCMH program showed significant benefits in terms of reduced mortality at 6 months, with similar costs and functional outcomes at 12 months. PCMH was not shown to improve all outcomes studied, but these results suggest that ongoing Medical Home management can have some benefit for patients without negatively impacting function or cost.

## Background

Hip fractures remain a major challenge to health care systems throughout the world [1], as both the number of hip fracture patients and the costs associated with their care increase over time [2-6]. Despite improvements in implant technology, morbidity and mortality rates in the first postoperative year remain high [7]. Numerous reports have shown that perioperative approaches that include medical optimization, multidisciplinary care and rehabilitation decrease immediate perioperative morbidity [8]. However, the significant morbidities and costs associated with hip fractures more often occur after discharge and are related to long-term management of medical comorbidities [9]. Further complicating matters, it is difficult to distinguish between morbidity related to the hip fracture itself as opposed to the overall frailty and vulnerability of the elderly population [10]. In theory, an optimal approach to managing patients would initially optimize the patient for surgery, match implants to the patients’ functional condition, and subsequently manage the patient’s overall health status including existing comorbidities. This multimodal approach has not been previously reported in the orthopaedic literature. If this approach successfully decreased readmissions and mortality, any increased costs associated with more aggressive post-operative management might be offset within a health system [11].

At the same time that hip fractures challenge the orthopaedic community, there are recognized shortcomings in hospital post-discharge transitions of care in general. As recently as 2009, half of Medicare fee-for-service patients who had a 30-day readmission did not visit their physician before being readmitted [12]. Communication gaps are suspected to account for a significant share of readmissions, and these occur between providers (e.g., discontinued prescriptions), between provider and patient (e.g., lack of patient education) and in transitions between the hospital and community setting [13-15]. Systematic reviews have reported significant reductions in 30-day readmission rates and cost savings associated with several enhanced discharge processes, most of which used enhanced in-hospital communication, payment incentives, and other multimodal approaches [16-18].

The Patient-Centered Medical Home (PCMH) model, which evolved from the Chronic Care Model [19], involves enhancing the role of primary care providers as the locus of care coordination. The PCMH is not a physical location, but a redesign of healthcare delivery to facilitate partnerships among an individual patient and his/her personal physician and family, all supported by resources including enhanced information systems and communications infrastructure. The American Academy of Pediatrics (AAP), American Academy of Family Physicians (AAFP), and other societies representing approximately 330,000 U.S. physicians describe 7 joint principles of the PCMH. These principles include each patient having an ongoing relationship with his/her personal physician, highly coordinated care, enhanced access to care, and payment structures that reflect the value of care management work [20]. At our health system, a PCMH model attempts to reduce post-discharge morbidity by using nurse case managers to coordinate the transition from hospital to home. Case managers use early telephone outreach, medication reconciliation, social support assistance, and ensure timely follow-up with primary care physicians in the month immediately following surgery.

We compared rates of mortality, hospitalizations, emergency department visits and prescription orders between two prospective cohorts of hip fracture patients, both managed with identical perioperative protocols and one group subsequently managed via PCMH. Costs, functional outcomes and quality of life were also studied as secondary outcomes in a smaller subsample. We hypothesized that the best outcomes would occur in patients managed initially with a standardized approach and subsequently with PCMH management.

## Methods

### Setting

This was a prospective, non-randomized two-cohort study conducted at two level-1 trauma centers within Geisinger Health System (GHS), a large integrated health system located in Pennsylvania. GHS serves a 41-county area with approximately 2.5 million people, employs a total of 800 physicians including 200 primary care physicians, and operates 41 outpatient clinics and five hospitals. The average duration of care for patients in the system exceeds ten years, and an electronic health record has been the sole-source ambulatory record for all patients since 2001. The Department of Orthopaedics also maintains a prospective trauma registry in which all treated fractures are recorded and classified according to the AO/Orthopaedic Trauma Association (AO/OTA) radiographic system.

Beginning in 2006, GHS implemented a “Patient-Centered Medical Home” (PCMH) program, referred to as “ProvenHealth Navigator®”. This program has been described in detail elsewhere [21,22]. This PCMH model represents a partnership between the health system and an affiliated insurance provider (Geisinger Health Plan) and is comprised of several parallel initiatives to improve overall quality of care. The primary element of interest for this study is that office-based nurse case managers (CMs) are hired by the insurance provider and integrated into the clinical care team at specific primary care sites. These CMs focus on coordinating services for at-risk patients who belong to that clinic, and they provide early outreach and close follow-up for patients who are transitioning home after a hospital discharge for any reason including hip fracture surgery [23]. Quality measurement and reporting encourage adherence to evidence-based care guidelines, and practices and individual physicians are paid additional stipends and can share in estimated savings, subject to meeting quality goals. No additional stipends at any site were tied directly to this particular study.

During the transition period of 2006–2011, the PCMH program was introduced to 41 clinic sites in phases. Although the order of site enrollment was not randomized, clinic sites were geographically dispersed and neither individual physicians nor patients could choose whether to participate in the PCMH program. Thus, the transition period provided an opportunity for a natural experiment to examine clinical outcomes of patients undergoing hip fracture surgery who could be categorized as either PCMH or non-PCMH according to the clinical site where their primary care physician was located. This study was approved by the Geisinger Institutional Review Board (IRB).

### Study population

All patients aged 60 or over who presented to either of two hospitals with an isolated closed, acute proximal femur fracture and were surgically treated from January 1, 2010 to June 30, 2011 were enrolled in this study. Acute fractures were defined as fractures sustained within 48 hours prior to presentation, and proximal femur fractures were defined as fractures diagnosed by plain radiography and classified as AO/OTA fractures 31-A (trochanteric) or 31-B (neck) by the attending surgeon on call. Patients were excluded if they sustained fractures that were open, high energy, related to osteomyelitis or neoplastic disease, or radiographically occult. Patients were also excluded if they presented in a moribund state, where any consideration of surgical treatment was deemed inappropriate, or if they elected to be treated non-operatively.

All patients received preoperative and perioperative care according to defined protocols that were jointly established by the two hospitals prior to the study (Table 1). After discharge, patients whose primary care physician belonged to a clinic site in the PCMH program were categorized as the “PCMH group”. These patients received follow-up care from a nurse case manager including: telephone follow-up within 48 hours; medication management (reconciliation and review of medications, timing and doses); home safety assessment; development of an action plan including who to notify about concerns; assistance with social supports and rehabilitation services (if necessary); weekly phone calls from an automated voice response system to inquire about adverse effects; additional follow-up calls for higher risk patients; and an in-person follow-up visit for the patient and family with the primary care physician and nurse case manager within 7 days. Patients whose primary care physician did not belong to an active PCMH site were categorized as “control” patients and received usual care.

**Table 1:** Overview of perioperative and post-discharge protocol for all patients in the study

**Perioperative management (All patients)**	**Post-discharge management (All patients)**	**Medical home management (Patient-Centered Medical Home patients only)**
1. Perioperative risk assessment and management	1. Follow-up with High-Risk Osteoporosis Clinic (if needed)	1. Nurse Case Manager (CM) makes initial call to patient within 24–48 hours of hospital discharge.
2. Timing of surgical intervention	2. Continuation of aggressive physical therapy	
3. Prophylactic antibiotics		
4. Thromboembolic prophylaxis	3. Deep venous thrombosis (DVT) Prophylaxis	2. CM reviews medication list with patient.
5. Prevention and management of delirium		
	4. Wound check, functional evaluation, and radiographic examination at 6 weeks	3. CM ensures that follow-up visit is scheduled with primary care provider within 7 days.
6. Prevention of decubitus ulcers		
7. Prevention of Constipation		
8. Physical therapy intervention	5. Periodic assessment (no less than every 3 months) until a baseline functional state or death occurs	4. CM ensures that a patient-specific action plan is in case if patients have any trouble.
9. Assessment for underlying osteoporosis		
10. Appropriate discharge placement		5. Patients receive weekly calls (2–3 minutes) for 4 weeks, to ask about complications or areas that need follow-up from CM.

Data were extracted from the electronic health record and insurance claims databases to record all deaths, hospitalizations, emergency department visits, and prescription orders during the 12 months following surgery. The health system receives monthly updates on mortality from Social Security for all patients, even those who no longer actively seek care in the system, so data on mortality is known to be 100% complete. For a secondary analysis of cost outcomes, we extracted insurance claims information on the subset of patients with a specific insurance provider (Geisinger). In addition, patients surviving at 12 months were contacted by telephone and asked to complete a quality-of-life (EQ-5D, or EuroQOL) questionnaire and the pain/function subsection of the Harris Hip Score questionnaire (Table 2) [24,25].

**Table 2:** Questions asked via telephone questionnaire to surviving patients at 12 months follow-up

**EQ-5D quality-of-Life (EuroQOL) index questions (respondent chooses one answer)**	**Harris hip score pain/function subsection (response choices not shown)**
**1. Mobility**	**1. Pain**
I have no problems in walking about.	How would you describe your hip pain?
I have some problems in walking about.	
I am confined to bed.	
**2. Self-care**	**2. Support**
I have no problems with self-care.	How much support do you need when walking?
I have some problems washing or dressing myself.	
I am unable to wash or dress myself.	**3. Distance Walked**
**3. Usual activities**	How much are you able to walk?
I have no problems with performing my usual activities.	
I have some problems with performing my usual activities.	
I am unable to perform my usual activities.	**4. Limp**
	Do you walk with a limp?
**4. Pain/Discomfort**	
I have no pain or discomfort.	**5. Shoes/Socks**
I have moderate pain or discomfort.	Are you able to put on your own shoes and socks?
I have extreme pain or discomfort.	
**5. Anxiety/Depression**	**6. Stairs**
I am not anxious or depressed.	Are you able to climb stairs?
I am moderately anxious or depressed.	**7. Public transportation**
I am extremely anxious or depressed.	Are you able to board a bus?
**6. Quality-of-life scale question**	**8. Sitting**
To help people say how good or bad a health state is, imagine a scale on which the best state you can imagine is marked 100 and the worst state you can imagine is marked 0. We would like you to indicate on this scale how good or bad your own health is today, in your opinion.	Are you able to sit comfortably?

### Statistical analysis

The primary endpoint for this study was all-cause mortality following hip fracture surgery, and so we used nonparametric survival analysis to compare survival between the two cohorts at 6 and 12 months. Secondary endpoints were rates of inpatient hospitalizations, emergency department visits, and prescription orders during the 6 and 12 months following surgery, which were compared using Poisson regression. Finally, as tertiary endpoints, we used Student-t testing to compare mean medical costs (pharmacy, non-pharmacy and total) at 6 and 12 months, and questionnaire scores among responders at 12 months. Costs were normalized to set the PCMH group’s mean monthly pharmacy cost to $100 in order to protect business-sensitive information. For all analyses, generalized estimating equation (GEE) methods were used to cluster observations to account for the matching of subjects described below. All statistical analysis was performed using SAS software (SAS 9.2, Cary, NC) with differences at p < 0.05 considered statistically significant.

This study was non-randomized, and although PCMH assignment depended only on the geography of the clinic sites and not individual patient/physician preference, we recognized there could still be unbalanced confounding variables in the two groups. We therefore limited the analyses to a matched set of cases and controls. Each PCMH patient (case) was matched to one non-PCMH patient (control) based on surgery date (±90 days) and a propensity score to adjust for disease severity following the methods of Rosenbaum and Rubin [26,27]. A logistic regression model calculated each patient’s propensity score (i.e., propensity for belonging to the PCMH group, on a scale of 0–1) using sex, age, Charlson Comorbidity Index, number of admissions and emergency department visits during the 12 months prior to surgery, and history of 10 comorbidities (hypertension, stroke, myocardial infarction, congestive heart failure, chronic kidney disease, other fractures, osteoporosis, anemia Alzheimer’s disease, and cancer). PCMH patients were excluded from analysis if they had propensity scores that did not overlap with any control patients, and cases were only matched to controls whose propensity scores fell within a tight caliper range in order to ensure balance between the cohorts [28]. This matching procedure was performed on the entire study population to generate two matched cohorts for analysis of mortality/utilization outcomes (for which all subjects had available data), and then repeated to generate matched subgroups for cost (where only some patients had claims data) and functional outcomes (where only a subset of patients had survey responses at 12 months). Because of our strict inclusion/exclusion criteria limiting the study only to patients with a specific fracture type and operative treatment, further details of the fracture type or surgery were not used for matching.

## Results

A total of 303 hip fracture patients met eligibility criteria for the study during the enrollment period, 108 of whom received PCMH post-discharge care and 195 of whom did not. The mean age was 81 years (range 60–100), and two-thirds (201 of 303) of patients were female. The overall survival rate at 12 months for this entire population was 219/303 (72%). After matching cases with controls, eleven PCMH patients had to be excluded for insufficient matches, resulting in a final study cohort of 97 PCMH cases matched to 97 non-PCMH controls. In the matched set, the mean age was 82 years (range 76–89) and 142/194 (73%) were female. Table 3 presents all baseline characteristics of the two primary matched cohorts (n = 194) for comparison.

**Table 3:** Baseline characteristics of the case and control cohorts, after 1:1 matching based on date of surgery and propensity scoring method

	**Medical home cases (n = 97)**	**Controls (n = 97)**
Age at surgery in years, Mean (SD)	82 (9)	82 (9)
% Male	28	26
Charlson comorbidity index, Mean (SD)	2.4 (2.1)	2.3 (2.2)
% Hypertension	76	78
% Stroke	19	14
% AMI	36	37
% Heart failure	22	22
% CVD	20	20
% Renal disease	28	29
% Diabetes	26	26
% Cancer	32	25
% Alzheimer’s	15	19

For the secondary analysis of healthcare costs, claims data were available for 66/108 (61%) of the PCMH patients and 42/195 (21%) of the non-PCMH patients. For the quality-of-life and pain/function telephone questionnaires administered to non-deceased patients at 12 months, the survey response rate was 41/82 (50%) for PCMH patients and 50/137 (37%) for non-PCMH patients. Repeating the matching procedure for the analysis of secondary outcomes yielded 15 matched pairs with cost data who survived 12 months (n = 30), and 35 matched pairs with pain/function data who survived 12 months (n = 70).

**Figure 1:**
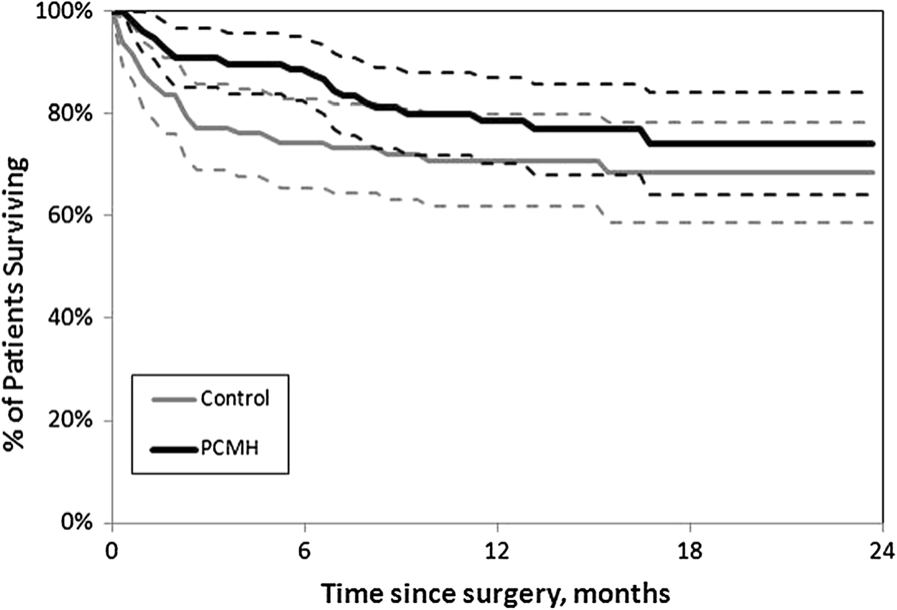
**All-cause mortality survival curves for “Patient-Centered Medical Home” (PCMH) and control cohorts.** Dashed lines around each curve represent 95% confidence intervals.

Table 4 presents a summary of all outcomes at 6 and 12 months after surgery. At 6 months post-operatively, patients receiving PCMH post-discharge management had a significantly lower mortality rate than patients receiving standard care (11 vs. 26% respectively, p < 0.01). At 12 months, a difference persisted (23 vs. 30%, p = 0.12), though it was no longer statistically significant. Figure 1 presents the all-cause mortality survival curves for these two cohorts. The mean Harris Hip Pain/Function Score was also significantly improved at 12 months in the PCMH group as compared to controls (73 vs. 64, p = 0.04). Otherwise, no differences between the cohorts reached statistical significance. Differences in numbers of all-cause hospitalizations, ED visits and prescription orders per patient were not significantly different at 6 months (p = 0.69-0.91) or 12 months (p = 0.16-0.83). The mean EQ-5D quality-of-life index was similar between the two groups (0.73 vs. 0.76, p = 0.49), as was the mean EQ-5D Scale. For those patients surviving 6 months, mean pharmacy and non-pharmacy costs were lower in the PCMH cohort but not significantly so (p = 0.27-0.95), and a similar trend persisted at 12 months (p = 0.20-0.45).

**Table 4:** Outcomes at 6 and 12 months for the two cohorts, including mortality, hospitalizations, emergency department (ED) visits, prescription orders, and costs, with significant differences in bold

	**Medical home cases**	**Controls**	**p-values**
*Mortality*			
N, subjects per group	97	97	--
N, Deaths at 6 months (%)	11 (11%)	25 (26%)	**<0.01**
N, Deaths at 12 months (%)	22 (23%)	29 (30%)	0.12
*Healthcare utilization*			
N, matched subjects per group	97	97	--
Hospitalizations per 100 patients at 6 months	12.4	13.4	0.84
ED visits per 100 patients at 6 months	35.1	34.0	0.91
Prescription orders per patient at 6 months	34.0	31.6	0.69
Hospitalizations per 100 patients at 12 months	23.2	25.7	0.83
ED visits per 100 patients at 12 months	69.6	54.3	0.42
Prescription orders per patient at 12 months	56.5	40.2	0.16
*Healthcare costs*^*†*^			
N, matched subjects per group	15	15	--
Mean pharmacy costs per patient-month (0–6 months)	$100	$175	0.27
Mean non-pharmacy costs per patient-month (0–6 months)	$3,527	$3,572	0.95
Mean total costs per patient-month (0–6 months)	$3,627	$3,509	0.89
Mean pharmacy costs per patient-month (0–12 months)	$69	$141	0.20
Mean non-pharmacy costs per patient-month (0–12 months)	$1,212	$1,452	0.45
Mean total costs per patient-month (0–12 months)	$1,281	$1,496	0.52
*Quality of life and function at 12 months*			
N, matched subjects per group	35	35	--
Mean EQ-5D index*	0.76	0.73	0.49
Mean EQ-5D QOL scale**	72	72	0.99
Harris hip pain/Function score^	73	64	**0.04**

Sample sizes differ among outcomes because of availability of cost, questionnaire data, and questionnaire outcomes were assessed only at 12 months.

*EQ-5D Index is on a scale of 0–1 with 1 = best.

**EQ-5D QOL Scale is on a scale of 0–100 with 100 = best.

^Harris Hip pain/function score is on a scale of 0–91 with 91 = best.

^†^All costs have been normalized such that pharmacy costs (0–6 months) for the MH group = $100.

## Discussion

Our study demonstrates, in a prospective study of patients receiving a unified in-hospital protocol, that the addition of post-discharge care from a “Patient-Centered Medical Home” program, including a nurse case manager for primary care-based follow-up, was successful in significantly reducing mortality in the 6 months following hip fracture and showed evidence of benefits to hip pain and function as well. While it may be unrealistic to expect that such a post-discharge program would eliminate mortality/morbidity, this study makes a promising case for utilizing PCMH programs for a focused, limited period following discharge. Although significant benefits in reduced admissions, emergency department visits or medication utilization and costs were not seen, the two cohorts remained similar in these respects, suggesting that the aggressive follow-up by the PCMH program did not substantially drive up healthcare utilization or costs. Future larger-scale studies should help to better define the optimal patient population for such programs and refinements that would provide further benefits for patients undergoing hip fracture surgery. We note that, while we found more published estimates of 1-year mortality than 6-month mortality, our control group's 6-month and 12-month mortality rates of 26 and 30%, respectively, appear to be consistently in the range of the published findings of Johnston et al. [29] and others who have followed operative treatment of hip fractures.

Mortality and morbidity after hip fracture in elderly patients remains a serious problem; however, there is growing evidence to suggest that patient factors and specific details of the approach to post-fracture treatment can impact mortality and quality of life. As noted by Johnston et al., many studies report mortality following hip fracture as a simple percentage and do not take into account age, gender and other factors [29]. In a recent systematic review, Butler et al. concluded that while neither implant type nor surgical approach were associated with differences in mortality rates, patient factors such as age, sex, pre-fracture functioning, and cognitive impairment were directly related to mortality and functional outcomes [30]. This literature suggested that the main mortality risk for elderly women is in the 6 weeks following surgery, while the risk for men is higher near 6 months, with comorbidities and age impacting different subgroups. In a study that complements our current results in a different population, Rahme et al. retrospectively reviewed 11,326 patients receiving hemiarthroplasty for hip fracture and showed that the small percentage of those (16%) who received post-discharge home care had a lower mortality rate at 3 months than those who received no home care [31]. While the data collected for this study do not allow us to pinpoint the exact reason why the PCMH program influenced the 6-month mortality rate in our study, we believe that by providing reinforcement during the critical 4 weeks following discharge, the primary care PCMH team may be able to better engage, activate and educate patients as well as identify and resolve issues that may otherwise be missed or caused by care gaps during the transition from hospital to home.

The main strengths of our study are its population and statistical design. We were able to use patients in an integrated health system where electronic health records were available for the hip fracture population to quantify mortality, inpatient admissions, emergency department visits and other healthcare utilization. Furthermore, we were able to take advantage of the fact that a PCMH program was in the middle of being implemented such that patients would naturally be assigned to either an intervention group (PCMH) or control group for post-discharge care. Finally, we employed robust statistical methods to identify and balance confounding variables in the two cohorts through matching. The chief limitations of the study are the modest sample size and the fact that it was a non-randomized study, though we took steps to address biases due to confounding through the matched study design. Nevertheless, not all subjects could be appropriately matched to controls, resulting in a further reduced sample size. We note, for example, that the number of deaths between 6 and 12 months was small, and with a larger sample size, some of the nonsignificant findings here may also have reached statistical significance. For the secondary outcomes of cost and functional questionnaires, we also note that not all subjects responded to the telephone questionnaire or had the appropriate insurance provider that allowed us to measure costs. Finally, the questionnaires were administered at 12 months only and we did not collect preoperative scores, though we expect that acute injury patients would have both EQ-5D and functional scores similar to the general population in their own age range, on average. While we took advantage of the data collection methods of an integrated health system (EHR tracking, claims data, survey center) to perform this investigation, we note that standardized protocols and PCMH models are increasingly being adopted across the U.S. and we believe that these results should be highly translatable to a variety of healthcare settings.

Increasingly, society, patients and payors are demanding measurable quality outcomes. The biggest social impacts may be made by improving the outcome of patient groups whose injuries significantly affect society, such as the elderly patients who sustain hip fractures. Given the multitude of different implant studies which have shown little difference in one-year outcomes for this common yet socially-disruptive injury, studies like this one suggest that the patient care that takes place outside the operating room may ultimately have the biggest impact on improved outcomes. The biggest improvements thus far in hip fracture care have not come from changes in implant technology (e.g., switching to a locked side plate or to an intramedullary device) [30], but from multidisciplinary management of these patients in the acute setting; longer-term benefits of these in-hospital interventions, however, have not been widely demonstrated [32-34]. It is our hope that the current study provides new evidence regarding important patient outcomes and costs, comparing an intrinsic control group with an experimental group, and that future work will support compelling arguments to government and private payors that the most cost-effective standard of care also gives the highest quality outcome. We recognize that the majority of care-coordination efforts thus far have focused on chronic diseases such as congestive heart failure or diabetes, and that evidence of effectiveness has been mixed, depending on the specific features of the intervention [35-38]. Future work should determine which specific conditions yield high-value results in order to avoid the error of a one-size-fits-all approach.

## Conclusion

Patients receiving aggressive post-discharge care from a “Patient-Centered Medical Home” program showed significant benefits in terms of reduced mortality, improved function, and trends toward reduced medical costs in the time period following hip fracture. Future studies with larger cohorts of PCMH patients are highly encouraged in order to reinforce these results and further examine the details of how PCMH may or may not benefit individual patients. Post-surgical care of elderly, multi-comorbid patients is complex, but these results suggest that ongoing PCMH management can benefit patients in the critical period immediately following a hip fracture and surgery.

## Consent

A HIPAA waiver of authorization and consent was granted for the retrospective review of records for this study. A partial waiver of authorization and consent was granted for prospectively identifying patients for follow-up questionnaires over the telephone, and completing the telephone survey implied verbal consent on part of the patient.

## Competing interests

The authors declare that they have no competing interests.

## Authors’ contributions

JHG contributed in the study concept and design, data analysis, and preparation of the manuscript. TRB contributed in the study concept and design and preparation of the manuscript. KAS contributed in the data collection and preparation of the manuscript. KI contributed in the preparation of the manuscript and critical review. WRS contributed in the study concept and design, preparation of the manuscript, and critical review. All authors read and approved the final manuscript.
